# Virtual Reality in Obesity Management: Addressing Key Limitations in Clinical Application

**DOI:** 10.2196/68269

**Published:** 2025-06-27

**Authors:** Xin Hu, Bin Wei

**Affiliations:** 1 The 1st Affiliated Hospital, Jiangxi Medical College, Nanchang University, 17 Yongwai Zheng Street, Nanchang, 330000, China, 86 15072518856

**Keywords:** obesity, virtual reality, psychological treatment, embodiment, motivational interviewing, self-conversation

We are writing to express our appreciation for the recent publication in the *Journal of Medical Internet Research* titled “Assessing the Clinical Efficacy of a Virtual Reality Tool for the Treatment of Obesity: Randomized Controlled Trial” [1]. The authors propose an innovative approach to obesity treatment that leverages the interactivity and immersive experience of virtual reality (VR) technology through a randomized controlled trial, providing new scientific evidence for obesity management. We commend the meticulous work and significant contributions of this study. However, while the randomized controlled design enhances the validity of these findings, there are several limitations worth further consideration.

First, in the VR experience, the system may not accurately simulate individual body types and postural characteristics, particularly for participants with significant body shape differences. This discrepancy can lead to a misalignment between the virtual representation and the participants’ self-perception, making it challenging for them to fully immerse themselves in their self-role or envisioned future selves. Furthermore, when individuals with obesity encounter substantial differences between their actual post–weight loss appearance and their anticipated image through the VR experience, it may induce feelings of anxiety or depression, ultimately undermining their confidence in future weight management goals. Thus, this cognitive dissonance could diminish the positive impact of the intervention, hindering participants from achieving the desired behavioral and psychological transformations.

Second, the use of VR tools imposes a high technical proficiency requirement on participants. Although the study included participants with basic digital skills, individuals who are less familiar with digital technology, particularly older adults, rural residents, or those from cultural backgrounds with limited exposure to digital devices, may experience difficulties in adapting to and accepting VR equipment [2]. This could lead to a dropout of potential participants due to technical barriers, ultimately affecting the actual effectiveness of the VR intervention. Future studies should consider incorporating adaptive training, technical support, and multicultural considerations to ensure the effectiveness and broader applicability of VR tools among diverse populations.

Moreover, while the article discusses the recruitment and randomization process of participants, it lacks detailed information on whether the sample represents the broader population of individuals with obesity [3]. This may limit the generalizability of the findings. Additionally, although the study mentions short- and medium-term follow-ups, it does not address a long-term follow-up, which is essential for evaluating the sustainability of the intervention effects and whether participants can maintain the benefits of treatment over time.

In summary, VR holds promise as an important psychological intervention tool for treating obesity. The authors’ innovation and insights into VR treatment for obesity are commendable. However, these limitations may affect the interpretation and dissemination of the study results, emphasizing the need for caution when assessing the clinical efficacy of VR tools in obesity management.

## Abbreviations

VR: virtual reality
